# Enhanced Expression of miR-34a Enhances *Escherichia coli* Lipopolysaccharide-Mediated Endometritis by Targeting LGR4 to Activate the NF-*κ*B Pathway

**DOI:** 10.1155/2021/1744754

**Published:** 2021-08-27

**Authors:** Xiaofei Ma, Baoyi Yin, Shuai Guo, Talha Umar, Junfeng Liu, Zhimin Wu, Qingqing Zhou, Arshad Zahoor, Ganzhen Deng

**Affiliations:** ^1^Department of Clinical Veterinary Medicine, College of Veterinary Medicine, Huazhong Agricultural University, Wuhan, China; ^2^College of Animal Science, Tarim University, Alar, Xinjiang, China; ^3^College of Veterinary Sciences, The University of Agriculture Peshawar, Pakistan

## Abstract

**Background:**

Persistent endometritis caused by bacterial infections has lethal effects on the reproductive performance of dairy cattle, which compromises animal welfare and delays or prevents pregnancy. The microRNA (miRNA) miR-34 family plays a pivotal role in the inflammatory process; however, the precise mechanism of miR-34a in endometritis has not been thoroughly elucidated to date.

**Methods:**

In this study, the endometrium of cows diagnosed with endometritis was harvested for bacterial culture and Gram staining to evaluate bacterial contamination of the uterus. Based on this, a bovine endometrial epithelial cell (BEND) inflammation model and a mouse model stimulated with lipopolysaccharide (LPS) *in vitro* and *in vivo* were constructed. Cell viability was assessed by CCK-8, trypan blue staining, and flow cytometry. H&E was applied to histopathological analysis. Immunohistochemical, immunofluorescence, qRT-PCR, and western blot assays were performed to measure the mRNA and protein expression of relevant genes. Online databases, plasmid construction, and dual-luciferase reporter gene assays were used to predict and validate the interaction between miR-34a and its target gene LGR4. Finally, mice were injected vaginally with a local antagomir to validate the role of miR-34a in murine uterine inflammation.

**Results:**

In this study, we observed that Gram-negative bacteria, represented by *Escherichia coli*, are the predominant pathogenic agents responsible for the recurrent occurrence of endometritis in dairy cows. Further, miR-34a was found to repress the expression of LGR4 by targeting the 3′ untranslated region (3′UTR) of LGR4. miR-34a was upregulated in bovine uterine tissues and bovine endometrial epithelial cells stimulated with LPS. miR-34a induced the release of the proinflammatory cytokines IL-1*β*, IL-6, and TNF-*α* by activating the phosphorylation of NF-*κ*B p65. Furthermore, IL-1*β* upregulated miR-34a transcription and downregulated LGR4 expression in an IL-1*β*-dependent manner.

**Conclusions:**

Taken together, our study confirmed that miR-34a is regulated by IL-1*β* and suppresses the level of the LGR4 3′UTR, which in turn exacerbates the inflammatory response. Thus, the knockdown of miR-34a might be a new direction for the treatment of endometritis.

## 1. Background

Persistent endometritis is one of the most common and serious concerns observed in dairy cows. Data from the United Kingdom suggest that approximately 10-15% of cows develop endometritis [1]. This disease has deleterious effects on the subsequent reproductive performance of cows, including calving numbers and milk yield [2]. Researchers have confirmed that excessive intrauterine infection mediated by pathogenic bacteria, such as *Staphylococcus aureus* and *Streptococcus*, and their products, such as lipopolysaccharide (LPS), frequently damage the endometrium [3], trigger endometritis [4], delay uterine regeneration [5], and perturb embryo implantation and survival [6]. A normal and active uterine defence mechanism is one of the most critical factors for the elimination of bacterial infection and postpartum recovery [2, 7]. It has been reported that antibiotics (local or systemic), disinfectants, sulfonamides, and hormonal therapy do not significantly improve the recovery rate of endometritis or pregnancy in cattle. Therefore, it is essential to understand the precise mechanism of endometritis in cattle.

MicroRNAs (miRNAs) are a type of small noncoding endogenous RNA, 19-25 bp in length [8], that downregulate gene expression at the pre-, intra-, and posttranscriptional levels by complementarily pairing with the 5′ or 3′ UTRs of target genes [8, 9]. It is highly species-conserved and has a spatiotemporally specific expression [10]. Many investigations have confirmed that microRNAs are involved in various physiological and pathological processes [11, 12], such as cell development [13], cell apoptosis, and cell proliferation [14]. The miR-34/449 family is conserved in mammalian organisms and generally consists of six homologous genes, namely, miR-34a, miR-34b, miR-34c, miR-449a, miR-449b, and miR-449 [15], among which miR-34a was the first identified [16]. Increasing evidence shows that miR-34a is an essential regulator of inflammatory responses [17, 18]. Simultaneously, NF-*κ*B-driven miR-34a has been found to impair the Treg/Th17 balance in autoimmune diseases [19]. Although expression of miR-34a in the uterine tissue of dairy cows has been reported, its specific function during endometritis is still unclear. Based on this information, we proposed a scientific hypothesis that miR-34a may also be involved in the regulation of endometrial inflammation.

Leucine-rich repeat-containing G protein-coupled receptor 4 (LGR4), also known as GPR48, is a member of the G protein-coupled receptor family [20]. In recent years, LGR4 has attracted much attention for its potential as a pharmacological target. LGR4, which is expressed in human and mouse placenta, uterus, ovary, and fallopian tube epithelium [21, 22], transduces signals through both the G protein-coupled signalling pathway and the Wnt signalling pathways [23] and has been found to be involved in the regulation of cell proliferation, differentiation, cell apoptosis, and cell migration [24]. Particularly, LGR4 has been implicated in inflammatory bowel disease [25] and embryonic development [26]. In addition, numerous studies have identified that innate immunity is overactivated in LGR4-deficient mice, which is considered to be a negative regulator of TLR2 and TLR4-related immune responses [27]. However, the specific mechanism of action by which LGR4 regulates the LPS-induced inflammatory responses has not been reported and needs further investigation. In this study, we explored the expression pattern of LGR4 in the uterus of infected cattle to further elucidate the precise molecular mechanisms that regulate endometrial inflammation.

Based on the aforementioned findings, we hypothesized that miR-34a may interact with LGR4 to modulate the LPS-induced inflammatory responses. In the present study, we investigated the expression patterns and the regulatory mechanism of its downstream effects in bovine endometrial epithelial cells (BENDs).

## 2. Results

### 2.1. Gram Staining for Assessment of Bacterial Contamination in the Endometrium of Cows

**Figure 1:**
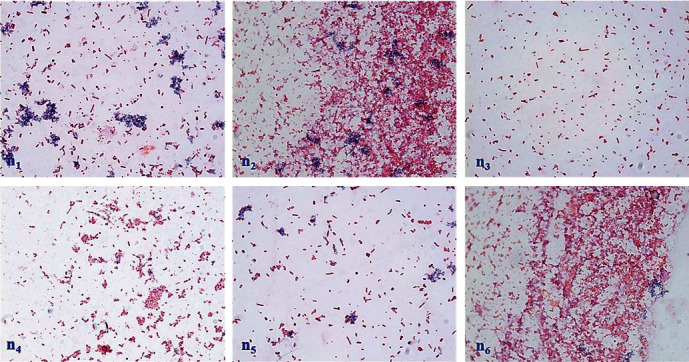
Gram staining for assessment of bacterial contamination in the endometrium of cows. Gram staining to identify bacterial contamination in the uterus of cows with endometritis (oil, ×100). Red represents Gram-negative bacteria, and blue represents Gram-positive bacteria. “*n*” indicates the number of detected cows (*n* = 6).

Bacteriological examination confirmed the presence of fungal septicemia and *Escherichia coli* as the main pathogens responsible for endometritis in cattle, often isolated from the uterus [4, 28, 29]. LPS is an endotoxin found in the outer cell wall of Gram-negative bacteria [30, 31]. To assess bacterial contamination in uterine tissue, Gram staining was conducted to examine the status and morphological feature of bacteria in the endometrium of uterine horn (*n* = 8) diagnosed with endometritis or pyometra. Gram staining (Figure 1) displayed that *E. coli* was identified in the uterine horns of cows diagnosed with endometritis. Cows with endometritis showed not only *E. coli* but also other Gram-negative and a few Gram-positive strains of bacteria. Consequently, we inferred that LPS of *E. coli* is the main causative agent of bovine endometritis.

### 2.2. Expression of LGR4 in Cow Uterus Tissues

**Figure 2:**
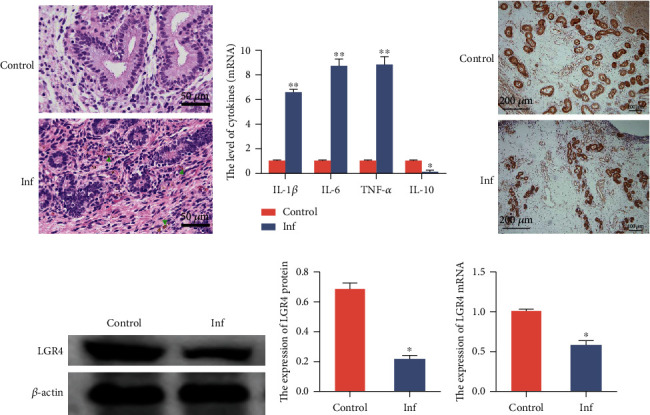
The expression of LGR4 in cow uterus tissues. (a) H&E pathology of cow uterine tissues (HE, ×200), scale bar = 50 *μ*m. (b) The mRNA levels of the inflammatory cytokines IL-1*β*, IL-6, TNF-*α*, and IL-10 detected by qRT-PCR to assess the extent of inflammation in uterine tissue. (c) The expression and location of LGR4 in uterine tissue by hematoxylin-eosin staining (H&E, ×100), scale bar = 200 *μ*m. (d, e) The expression of LGR4 protein was determined by western blot, and its level was quantified by IPP 6.0. *β*-Actin was used as an internal control. (f) The LGR4 mRNA was analysed by qRT-PCR. GAPDH was used as a control. Control represents the control group, and Inf represents the naturally infected cow uterine tissue. Data are expressed as the means ± SEM of three independent experiments (*n* = 20). ^∗^*P* < 0.05 and ^∗∗^*P* < 0.01 compared with the control group (Student's *t*-test).

Endometritis leads to endometrial injury and early loss of embryos [32]. Uteruses were collected from normal cows and from cows with endometritis. Firstly, changes in the endometrium were identified by H&E staining and histological score of activity. From their microscopic observations (Figure 2(a)) and scoring results (Table S4), the uterine tissues of infected cows revealed apparent inflammatory damage, with a large amount of inflammatory cell infiltration and visible swelling. qRT-PCR results showed that the endometritis tissues secreted the proinflammatory cytokines IL-1*β*, IL-6, and TNF-*α*, but there was a decreased secretion of IL-10, an anti-inflammatory mediator (Figure 2(b)). To confirm the expression of LGR4 in bovine uterus, immunohistochemical staining revealed that LGR4 was mainly present in the plasma membrane, extracellular lumen, cytoskeleton, and nucleus (Figure 2(c)). Moreover, the protein expression level of LGR4 determined by western blot and the mRNA level determined by qRT-PCR were decreased in the infected uterine tissues relative to the control uterine tissues (Figures 2(d)–2(f)). Thus, the expression of LGR4 was reflected to be downregulated in the presence of uterine inflammation, suggesting that LGR4 may be involved in the inflammatory response of the endometrium in cows.

### 2.3. LGR4 Suppresses the Secretion of Inflammatory Cytokines in BENDs

**Figure 3:**
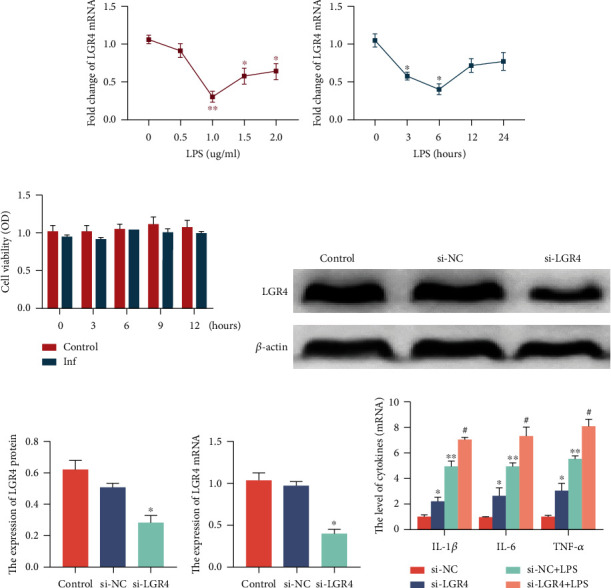
LGR4 suppresses the secretion of inflammatory cytokines in BENDs. (a) BENDs were treated with LPS (0, 0.5, 1.0, 1.5, and 2.0 *μ*g/ml) for 6 h. The LGR4 mRNA levels were analysed. (b) Cells were stimulated with LPS (1.0 *μ*g/ml) at 0, 3, 6, 12, and 24 h. The expression level of LGR4 was measured by qRT-PCR. (c) The effects of LPS (1.0 *μ*g/ml) on the viability of BENDs. Cell viability was determined with a CCK-8 assay kit. (d, e) Western blot analysis of LGR4 protein in BENDs after treatment with si-LGR4 or negative control (si-NC). *β*-Actin was used as an internal control. (f) qRT-PCR analysis of LGR4 mRNA expression in BENDs after treatment with si-LGR4 or negative control (si-NC). GAPDH was used as a control. (g) Cells were transfected with si-LGR4 or si-NC for 24 h and then stimulated with LPS (1.0 *μ*g/ml) for 6 h. The production of IL-1*β*, IL-6, and TNF-*α* was measured with qRT-PCR. GAPDH was used as a control. The data are presented as the means ± SEM of three independent experiments (*n* = 3). ^∗^*P* < 0.05 and ^∗∗^*P* < 0.01 versus the si-NC group; ^#^*P* < 0.05 and ^##^*P* < 0.01 versus the si-NC and LPS groups (cells transfected with si-NC after stimulation with LPS) (Student's *t*-test).

Given the above results, we further investigated whether the reduction in LGR4 induces the secretion of a number of inflammatory cytokines. BENDs were treated with different doses of LPS (0, 0.5, 1.0, 1.5, and 2.0 *μ*g/ml) at different time points (0, 3, 6, 12, and 24 h). The results indicated that LGR4 was decreased in a dose-dependent (Figure 3(a) and Figure S1) and time-dependent (Figure 3(b)) manner. We further analysed the effect of LPS on BENDs viability using CCK-8. BEND cell viability was found to be almost unaffected by LPS (1.0 *μ*g/ml) stimulation, as detected by CCK-8 assay (Figure 3(c)) and trypan blue exclusion test (under light microscope) (Figure S2). On the other hand, the effect of LPS treatment on apoptosis and cell cycle progression of BENDs at different times (0 h, 6 h, 9 h, and 12 h) was examined by flow cytometry, and it was found that LPS at 1.0 *μ*g/ml had little effect on cell cycle progression and cell viability (Figure S3), which is consistent with the above results. Furthermore, si-LGR4 or si-NC was transfected into cells to knock down LGR4 expression, and the transfection efficiency was verified by western blot (Figures 3(d) and 3(e)) and qRT-PCR (Figure 3(f)). Moreover, we found that knockdown of LGR4 significantly upregulates the secretion of the proinflammatory cytokines IL-1*β*, IL-6, and TNF-*α* by qRT-PCR (Figure 3(g)).

### 2.4. LGR4 Inhibits the Inflammatory Response by Blocking NF-*κ*B Phosphorylation

**Figure 4:**
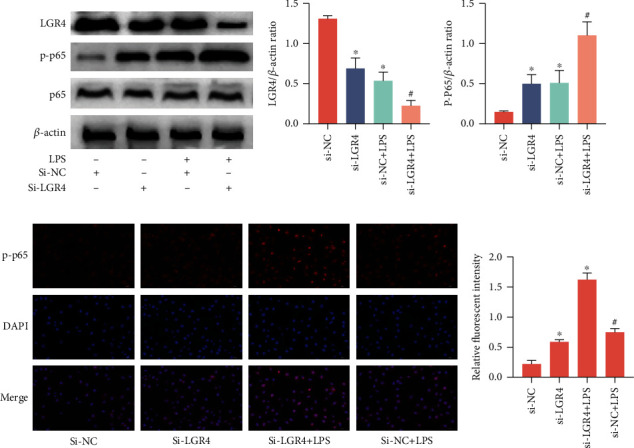
LGR4 inhibits the inflammatory response by blocking NF-*κ*B phosphorylation. Cells were transfected with 200 nM si-LGR4 or si-NC for 24 h and then treated with LPS (1.0 *μ*g/ml) for 6 h. (a) The expression of LGR4 and phosphorylated NF-*κ*B p65 in BENDs was determined by western blot. (b, c) The levels of LGR4 and phosphorylated NF-*κ*B p65 were determined by IPP in BENDs. (d, e) Translocation of the p65 subunit from the cytoplasm into the nucleus was evaluated by immunofluorescence. Blue spots represent cell nuclei, and green spots represent p-p65 staining. Data are presented as the means ± SEM of three independent experiments (*n* = 3). ^∗^*P* < 0.05 versus the si-NC group; ^#^*P* < 0.05 versus the si-NC and LPS groups (cells transfected with si-NC after stimulation with LPS) (Student's *t*-test).

LGR4/GPR48is a negative regulator of TLR2/4-associated immune responses [27]. Therefore, we hypothesize that LGR4 negatively regulates the activation of NF-*κ*B p65 phosphorylation and suppresses the transcription of proinflammatory cytokines to alleviate inflammation. To verify the above hypothesis, the protein expression of LGR4 and phosphorylation of NF-*κ*B p65 were detected by western blot after silencing LGR4. The data revealed that the level of LGR4 protein was apparently decreased (Figures 4(a) and 4(b)); conversely, phosphorylation NF-*κ*B p65 increased (Figures 4(a) and 4(c)). Immunofluorescent staining also confirmed this result (Figures 4(d) and 4(e)). Taken together, these data confirmed that the knockdown of LGR4 significantly enhances the activity of phosphorylated NF-*κ*B p65.

### 2.5. MiR-34a Inversely Correlates with the Expression of LGR4

**Figure 5:**
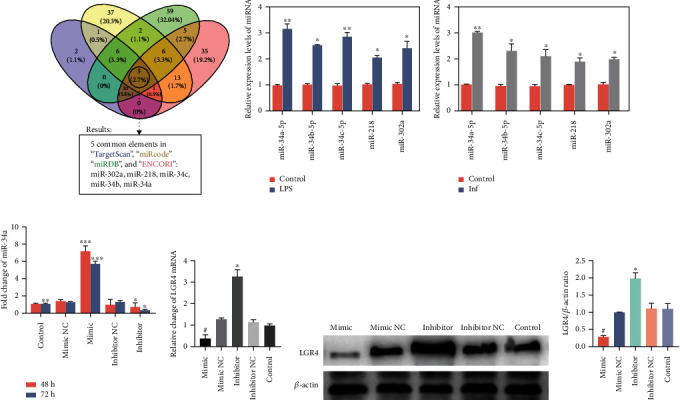
MiR-34a inversely correlates with the expression of LGR4. (a) The Venn diagram shows the predicted overlap of the microRNAs that target the cow and murine LGR4 3′UTRs by miRcode, miRDB, TargetScan, and ENCORI. (b, c) The levels of the putative microRNAs, miR-34a, miR-34a, miR-34c, miR-218, and miR-302a were detected in BENDs treated with LPS (1.0 *μ*g/ml) and cow uterus tissues by qRT-PCR. U6 snRNA was used as an endogenous control. (d) Cells were transfected with bovine miR-34a mimics, miR-34a inhibitor, or negative control (mimic NC or inhibitor NC) for 48 or 72 h, and then the miR-34a transcription levels were detected by qRT-PCR. (e, f) LGR4 mRNA and protein levels were determined by qRT-PCR and western blot, respectively. *β*-Actin was used as an internal control. Data are presented as the means ± SEM of three independent experiments (*n* = 3). ^∗^*P* < 0.05 and ^∗∗^*P* < 0.01 versus the control group (b–d); ^#^*P* < 0.05 versus the mimic NC group; ^∗^*P* < 0.05 versus the inhibitor NC group ((e and g) cells transfected with miR-34a mimic) (Student's *t*-test).

Recent studies have shown that miRNAs are involved in the embryonic development and the various biological processes of ruminants [33]. Based on the finding that LGR4 has anti-inflammatory properties, four online websites (miRcode, miRDB, TargetScan, and ENCORI) were used to predict the putative microRNA that may target LGR4. By determining the overlap of the prediction results of all websites, as shown in Figure 5(a), it was found that 5 microRNAs (miR-34a, miR-34b, miR-34c, miR-302a, and miR-218) have the potential to target LGR4.

Based on this result, qRT-PCR was performed to verify the level of putative microRNAs under LPS stimulation (1.0 *μ*g/ml) (Figure 5(b)) and in infected cow uterus tissues (Figure 5(c)). The findings indicated that miR-34a, miR-34b, miR-34c, miR-218, and miR-302a were upregulated; in particular, miR-34a was increased approximately 5-fold.

To further determine the regulatory relationship between miR-34a and LGR4, an miR-34a mimic or inhibitor was transfected into BENDs, which were then stimulated with LPS (1.0 *μ*g/ml) for 6 h. The transfection efficiency was verified by qRT-PCR (Figure 5(d)). Figures 5(e)–5(g) show that the expression of LGR4 mRNA and protein in the mimic group was markedly lowered, but the inhibitor group showed the opposite result. The data further showed that miR-34a may directly negatively regulate LGR4 mRNA and then inhibit its translational level, which also confirmed the interaction between miR-34a and LGR4.

### 2.6. MiR-34a Directly Targets the 3′UTR of LGR4

**Figure 6:**
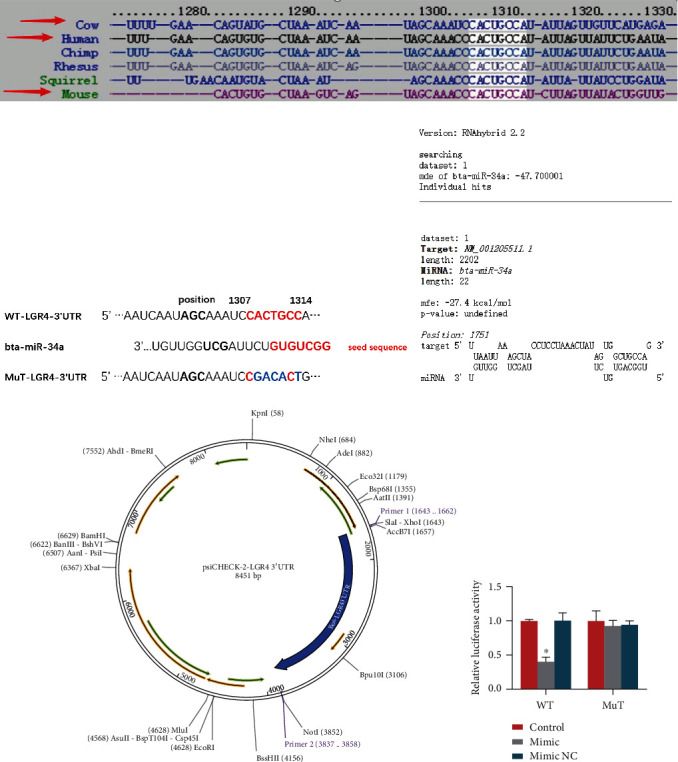
MiR-34a directly targets the 3′UTR of LGR4. (a) TargetScan predicts the interaction of miR-34 with the LGR4 among species. (b) Schematic of the design of the luciferase reporters with the wild-type WT-LGR4 3′UTR (WT) or the site-directed mutant-type MuT-LGR4 3′UTR (MuT). The nucleotides in red represent the “seed sequence” of miR-34a; the mutation nucleotides are marked in blue. (c) Alignment of the 3′UTR of LGR4 with miR-34a by RNAhybrid 2.2. (d) The psiCHECK-2 vector map (the insertion site of the LGR4 3′UTR is marked in light blue). (e) 293T cells were cotransfected with WT-LGR4 3′UTR or MuT-LGR4 3′UTR luciferase reporter vectors, together with miR-34a mimics or mimic NC (final concentration: 20 nM) as indicated. After 24 h, firefly luciferase activity was measured and normalized to Renilla luciferase activity. WT: wild type; Mut: mutant type. Data are presented as the means ± SEM of three independent experiments (*n* = 3). ^∗^*P* < 0.05 versus the control or the mimic NC group (Student's *t*-test).

LGR4 is a member of the leucine-rich repeat domain-containing G protein-coupled receptors, and it has been predicted that its interaction with miR-34a is highly conserved among species, as shown in Figure 6(a). Based on the website prediction results, miR-34a may target the 3′UTR of LGR4, and the interaction map is shown in Figure 6(b). Furthermore, RNAhybrid 2.2 also showed that miR-34a has the potential to bind the LGR4 3′UTR according to the calculation of minimum free energy (Figure 6(c)). To further explore the interaction mechanism between miR-34a and LGR4, LGR4 3′UTR (WT-3′UTR or MuT-3′UTR) were amplified and cloned into the psiCHECK-2 vector to construct the psiCHECK-2-LGR4 3′UTR plasmid (Figure 6(d)), which was then cotransfected with miR-34a mimics, NC mimics, or a negative control into 293T cells. The results of the dual-luciferase reporter gene assay in these cells demonstrated that the fluorescent activity of the WT-3′UTR was drastically decreased, while the MuT-3′UTR group showed no notable difference (Figure 6(e)), reflecting that miR-34a directly targets the 3′UTR of LGR4 mRNA.

### 2.7. MiR-34a Regulates Inflammation through NF-*κ*B in BENDs

**Figure 7:**
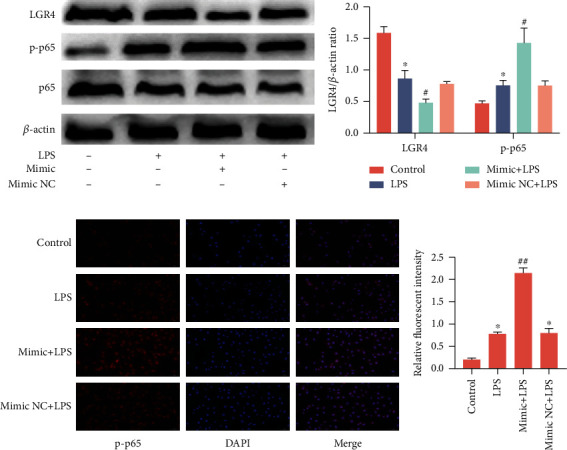
Overexpression of miR-34a promotes NF-*κ*B p65 translocation into the nucleus in BENDs. (a, b) Cells were transfected with miR-34a mimics or miR-34a mimic NC for 24 h, followed by exposure to LPS for another 6 h. The relative protein expression of LGR4 and NF-*κ*B p65 was measured by western blot. *β*-Actin was used as an internal control. (c) Immunofluorescence staining was performed to identify the expression of p-p65 (×400); scale bar = 50 *μ*m. Blue spots represent cell nuclei, and red spots indicate p-p65 staining. (d) The intensity of p-p65. Values are given as the means ± SEM of three experiments (*n* = 3). ^∗^*P* < 0.05 and ^∗∗^*P* < 0.01 versus the control (b and d) or the mimic and LPS (d) group; ^#^*P* < 0.05 and ^##^*P* < 0.01 versus the LPS group.

**Figure 8:**
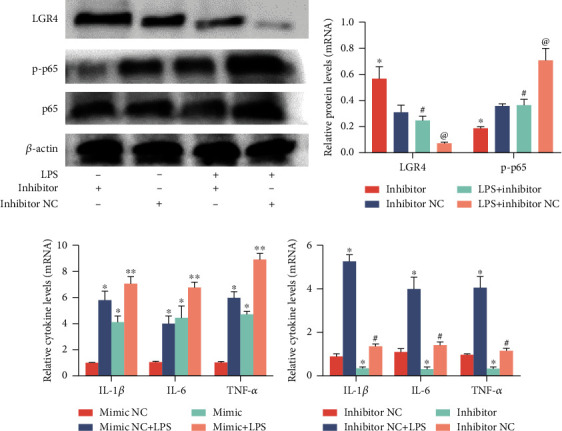
Inhibition of miR-34a suppresses inflammation in BENDs. (a) Cells were transfected with miR-34a inhibitor or negative control (inhibitor NC) for 24 h and then stimulated with LPS (1.0 *μ*g/ml) for 6 h. The relative protein expression of LGR4 and NF-*κ*B p65 was measured by western blot. *β*-Actin was used as an internal control. (b) Grey values of LGR4 and NF-*κ*B p65 protein were quantified by ImageJ software. (c, d) The secretion of IL-1*β*, IL-6, and TNF-*α* was detected by qRT-PCR. Values are given as the means ± SEM of three experiments (*n* = 3). ^∗^*P* < 0.05 and ^∗∗^*P* < 0.01 versus the inhibitor NC (cells transfected with miR-34a inhibitor NC) or mimic NC (cells transfected with miR-34a mimic NC) group; ^#^*P* < 0.05 versus the inhibitor group (b and d); ^@^*P* < 0.05 compared with the LPS and inhibitor group (b).

In this study, we determined that miR-34a was upregulated in both infected cow uterine tissue and endometrial epithelial cells stimulated by LPS (1.0 *μ*g/ml), contrary to the expression of LGR4. Considering the anti-inflammatory potential of LGR4, and to further explain the function of miR-34a in the inflammatory response, miR-34a gain-of-function and loss-of-function experiments were performed. The data showed that overexpression of miR-34a not only suppressed the expression of LGR4 (Figures 7(a) and 7(b)) but also promoted the phosphorylation of NF-*κ*B p65 (Figures 7(c) and 7(d)); conversely, inhibition of miR-34a significantly reduced protein levels of LGR4 and phosphorylation of NF-*κ*B p65 (Figures 8(a) and 7(b)). In addition, the production of IL-1*β*, IL-6, and TNF-*α* was much greater in the miR-34a mimic group (Figure 8(c)) but remarkably lower in the miR-34a inhibitor group (Figure 8(d)). Taken together, these results further implied that miR-34a has the potential to promote the progression of inflammation.

### 2.8. Inhibition of miR-34a Suppresses Endometritis in Mice

**Figure 9:**
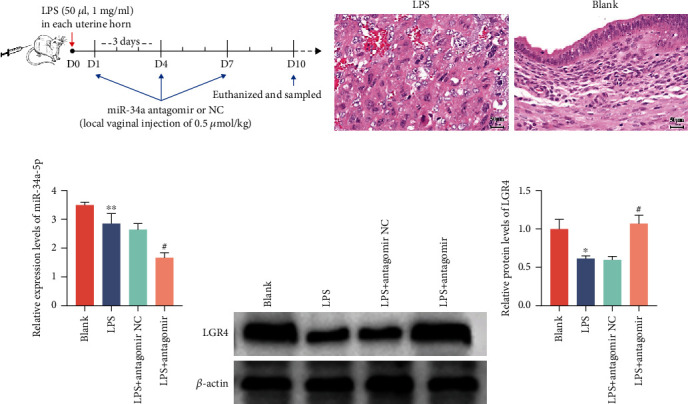
Inhibition of miR-34a enhances the expression of LGR4 in LPS-treated mice. (a) Schematic illustration of intrauterine injection of miR-34a antagomir or negative control. (b) H&E pathological analysis was conducted to identify a mouse model of endometrial inflammation (HE, ×200), scale bar = 50 *μ*m. (c) The mRNA level of miR-34a in mouse uterine tissues by qRT-PCR. (d, e) Western blotting was used to determine the LGR4 protein level. Grey values of the indicated proteins were measured by ImageJ software. Data are expressed as the mean ± SEM of three independent experiments (*n* = 3). ^∗^*P* < 0.05 and ^∗∗^*P* < 0.01 versus the blank group; ^#^*P* < 0.05 compared with the LPS and antagomir NC group. Comparisons among multiple groups were analysed by one-way analysis of variance with the Bonferroni posttest.

**Figure 10:**
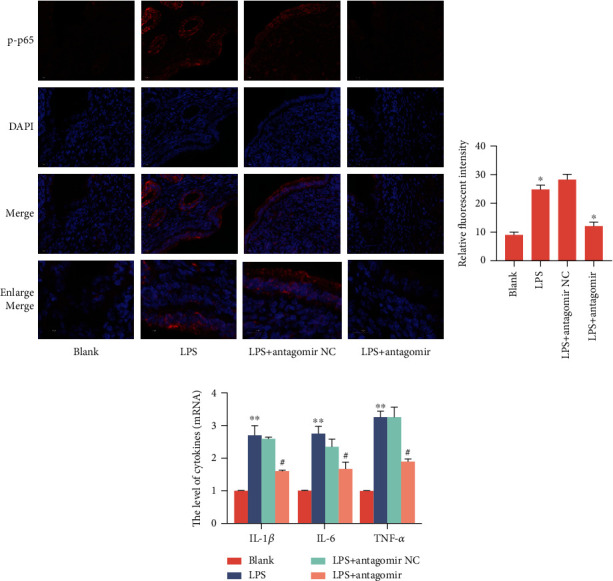
miR-34a antagomir attenuates NF-*κ*B activity and inflammatory cytokine secretion in mice. (a, b) Translocation of the p65 subunit from the cytoplasm into the nucleus was evaluated by immunofluorescence (×400). Blue spots represent cell nuclei, and red spots represent p-p65 staining; scale bar = 50 or 20 *μ*m. (c) Effect of *in vivo* injection of miR-34a antagomir on the mRNA levels of IL-1*β*, IL-6, and TNF-*α* in the uterus. Data analysis as in Figure 9.

miR-34a is highly conserved among species. To further investigate the role of miR-34a in endometritis *in vivo*, we established a mouse endometritis model following a previous laboratory method [34]. The groups were as follows: the blank group, the LPS group, the LPS+miR-34 antagomir NC group, and the LPS+miR-34a antagomir group. As shown in Figure 9(a), Kunming mice were infused with LPS (50 *μ*l, 1 mg/ml) (*n* = 18) in each uterine horn, and the day of injection was recorded as day 0 (D0) (LPS group). Control mice (*n* = 6) were injected with the same amount of phosphate-buffered saline (blank group). After 24 h, an miR-34a antagomir (*n* = 6) or antagomir NC (*n* = 6) (intrauterine injection of 0.5 *μ*mol/kg) [35] was administered to the LPS-treated uterus on D1, D4, and D7, and uterine tissues were collected on D10. Subsequently, H&E staining (Figure 9(b)) confirmed that LPS-stimulated mice caused endometritis with a large infiltration of inflammatory cells and edema in the inflamed uterine tissue compared to the normal group of mice. The qRT-PCR results (Figure 9(c)) verified the expression of miR-34a, showing a significant reduction after miR-34a antagomir treatment. Furthermore, LGR4 was remarkably inhibited after LPS stimulation, while its expression was upregulated after miR-34a antagomir injection (Figures 9(d) and 9(e)). Consistently, miR-34a antagomir suppressed the entry of NF-*κ*B into the nucleus (Figures 10(a) and 10(b)) and lowered the production of IL-1*β*, IL-6, and TNF-*α* (Figure 10(c)). Overall, our data revealed that knockdown of miR-34a can suppress inflammatory cytokines and thus alleviate endometritis.

### 2.9. IL-1*β* Suppresses LGR4 Expression by Enhancing miR-34a

**Figure 11:**
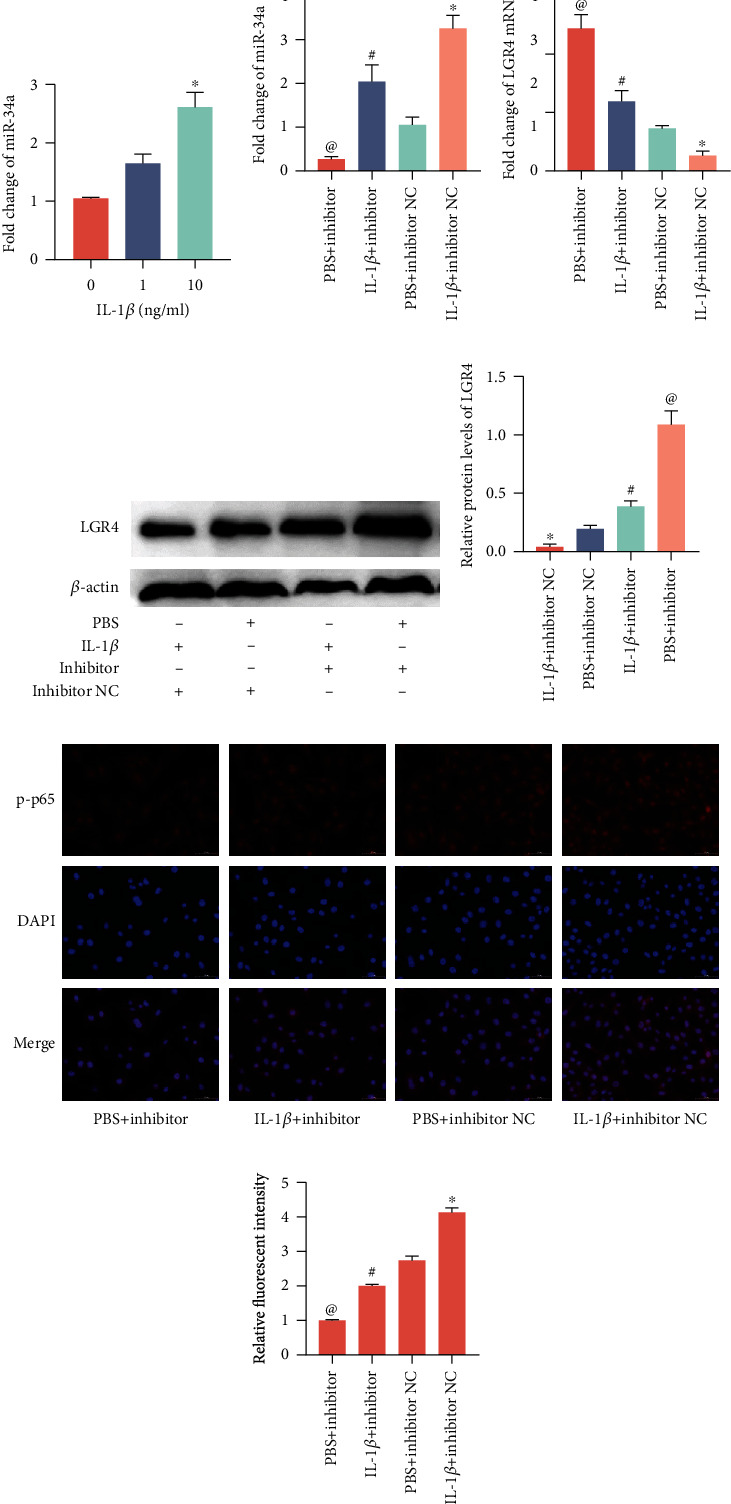
IL-1*β* suppresses LGR4 expression by enhancing miR-34a. (a) BENDs were treated with recombinant IL-1*β* (0, 1, and 10 ng/ml) for 12 h, and then miR-34a transcription levels were analysed by qRT-PCR. (b) Cells were transfected with miR-34a inhibitor or the negative control and then stimulated with recombinant IL-1*β* (10 ng/ml). The relative expression of miR-34a was normalized to U6 snRNA. (c) qRT-PCR assay was used to determine the LGR4 mRNA. (d) The expression of LGR4 and p-p65 was determined using western blotting. (e) Grey values of the indicated proteins were measured by ImageJ software. (f) Translocation of the p65 subunit from the cytoplasm into the nucleus was evaluated by immunofluorescence (×400). Blue spots represent cell nuclei, and red spots represent p-p65 staining; scale bar = 50 *μ*m. (g) The fluorescence intensity of p-p65. The integrated option density (IOD) of DAPI was used as an internal control. The IOD and area of cells were measured by IPP 6.0 software, and the fluorescence intensity of p-p65 was expressed as IOD/area. Data are expressed as the mean ± SEM of three independent experiments (*n* = 3). ^∗^*P* < 0.05 versus the inhibitor negative control group (untreated with IL-1*β*); ^#^*P* < 0.05 compared with the inhibitor group (untreated with IL-1*β*); ^@^*P* < 0.05 compared with the inhibitor group. Comparisons among multiple groups were analysed by one-way analysis of variance with the Bonferroni posttest.

Xie et al. [19] have shown that IL-6 and TNF-*α* activate p65 to bind to the miR-34a promoter and promote its transcription to enhance its activity. Therefore, we speculate that LPS induced the high expression of miR-34a in BENDs, which is most likely caused by the regulation of the proinflammatory cytokine IL-1*β* induced by activated NF-*κ*B p65. To evaluate the effect of IL-1*β* on the expression of miR-34a and LGR4, different concentrations of recombinant IL-1*β* (0, 1, 5, and 10 ng/ml) were used to treat BENDs. As shown in Figure 11(a), miR-34a was enhanced by incubation with IL-1*β* for 6 h in a dose-dependent manner (0, 1, and 10 ng/ml). Moreover, BENDs were transfected with an miR-34a inhibitor in the presence or absence of 5 ng/mL IL-1*β* treatment to further investigate the effect of IL-1*β* on miR-34a and LGR4. The qRT-PCR results (Figure 11(b)) showed that downregulation of miR-34a by the inhibitor was partially reversed by IL-1*β* stimulation. As we expected, the mRNA and protein expression of LGR4 was notably aggravated by the miR-34a inhibitor, which was rescued by incubation with IL-1*β*, as evidenced by Figures 11(c)–11(e). The immunofluorescence results showed that IL-1*β* induced NF-*κ*B p65 translocation into the nucleus, while silencing miR-34a apparently rescued this change (Figures 11(f) and 11(g)). Consequently, these above data together implied that IL-1*β* is an inducer of miR-34a enhancement, which can also mediate LGR4 expression.

**Figure 12:**
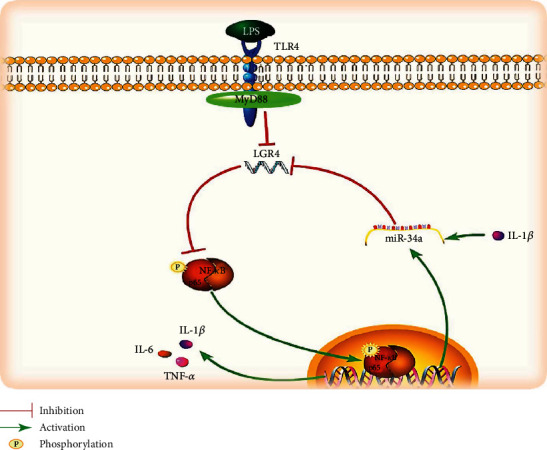
Schematic diagram depicting the signalling pathways for miR-34a in the regulation of LPS-triggered inflammatory responses in bovine endometrial cells.

In summary, our results demonstrated that IL-1*β* mediates the transcription of miR-34a and further positively regulates the secretion of inflammatory mediators via the LGR4-NF-*κ*B pathway, causing an excessive inflammatory response, which in turn damages the endometrium (as shown in Figure 12).

## 3. Discussion

Embryo loss caused endometritis is a key element in low calving rates on farms [36]. Bacterial infections are the most common cause of postpartum endometritis in dairy cows [37] and are responsible for failure in embryo implantation and growth of embryos [38]. To date, antibiotics have been universally accepted as a clinical approach to endometritis. However, antibiotic treatment can lead to severe drug residues and drug resistance. Therefore, it is critical to understand the specific pathogenesis of this disease and to discover new therapeutic approaches. MicroRNAs are prominently expressed in LPS-induced endometrial inflammation [28, 39]. However, the exact mechanisms in microRNA upstream and downstream regulation during endometrial inflammation remain to be elucidated. Here, our study demonstrated that miR-34a is a conserved repressor of LGR4 and is involved in the pathogenesis of endometriosis by inducing the release of inflammatory factors through the LGR4-NF-*κ*B axis. Additionally, we confirmed that the release of IL-1*β*, an inflammatory mediator, increases the expression of miR-34a and downregulates LGR4, forming a positive regulatory chain that causes further deterioration of the inflammatory response.

As expected, we found that LGR4 is a negative regulator that inhibits inflammation via the NF-*κ*B signalling pathway. However, the expression of LGR4 was decreased in the uterine tissues of naturally infected cows. To further elucidate the potential role of LGR4 in LPS-treated cells, si-LGR4 was transfected into BENDs. Our results suggest that knockdown of LGR4 activates phosphorylation and nuclear transcription of downstream NF-*κ*B p65, which in turn induces the secretion of inflammatory mediators. Thus, LGR4 may suppress inflammation, which is consistent with Wu et al.'s findings that LGR4 regulates the inflammatory response in keratinocytes [40].

Moreover, we also predicted that microRNAs target the bovine and murine LGR4 3′UTR and combined the results from four recognized databases: miRcode, miRDB, TargetScan, and ENCORI. The prediction results showed that miR-34a, miR-34b, miR-34c, miR-302a, and miR-218 were most likely to be target molecules that bind to the LGR4 3′UTR. Next, we determined the differences in expression levels of the five microRNAs by qRT-PCR, and finally identified miR-34a. Interestingly, among the five microRNAs, miR-34a was found to be the most notably upregulated. The relationship between miR-34a and LGR4 was then confirmed by the dual-luciferase reporter assay, which showed that miR-34a dramatically lowered the activity of the LGR4 3′UTR. Similarly, it has been recently reported that LGR4 is a common target of miR-34a and miR-34c in mice [32]. In conjunction with the results of the above study showing that LGR4 suppresses the production of proinflammatory mediators, we subsequently transfected BENDs with an miR-34a mimic or inhibitor to carry out gain-of-function and loss-of-function experiments to further investigate whether there was a suppressive effect of miR-34a on LGR4. Our data revealed that miR-34a indeed repressed the level of LGR4 mRNA and increased the transcription of NF-*κ*B p65 into the nucleus. We concluded that elevation or lowering of LPS can enhance or weaken the inflammatory response by altering the phosphorylation level of NF-*κ*B p65 and modulating the release of inflammatory cytokines, thereby impairing the expression of LGR4.

Collectively, these data suggest that the miR-34 family plays a pivotal role in autoimmune and other inflammatory responses [41, 42]. Most interestingly, NF-*κ*B p65 has been reported to promote miR-34a transcription, suggesting that chemokines or inflammatory cytokines produced by NF-*κ*B induction may target the promoter that binds miR-34a, thereby mediating miR-34a transcription and affecting the expression of downstream targets [43].

Consistent with the present findings, it has been reported that miR-34a aggravates wound inflammation [40]. Nevertheless, it is worth noting that studies have shown that enhancing miR-34a expression may hold promise in anti-inflammatory drug development [17], confirming the spatiotemporal expression specificity of microRNAs. It has been suggested that miR-34a may be regulated by upstream molecules, such as long noncoding RNAs, transcription factors, and proinflammatory mediators. Furthermore, it may be related to the regulation of multitargeted networks of miRNAs, indicating the need to search for molecules located upstream of miRNAs and investigate their specific regulatory mechanisms.

Finally, we aimed to elucidate the upstream elements regulating miR-34a upregulation. p53 has been shown not only to regulate miRNAs, including miR-34a/b/c, at the transcriptional level, but also to control the processing and maturation of specific miRNAs [44–46]; its role has been shown to be primarily in tumour suppression. There are few reports on the upstream regulators of miR-34a in the inflammatory response. Fortunately, IL-6 and TNF-*α* have recently been shown to regulate miR-34a transcription and participate in rheumatoid arthritis [19].

Sustained activation of NF-*κ*B can increase the expression levels of proinflammatory cytokines, including IL-1*β*, IL-6, and TNF-*α*. Among these, IL-6 and TNF-*α* are multifunctional proinflammatory mediators that play an important role in the development of chronic inflammatory responses. Importantly, IL-1*β* is a crucial mediator of the inflammatory response, which is essential for the host response and resistance to pathogens [47]. Therefore, we chose IL-1*β* as a candidate to investigate its effect on miR-34a transcriptional activity. The study data showed that IL-1*β* significantly promoted miR-34a transcription and suppressed LGR4 expression. In the future, more research should be performed on the specific mechanism of IL-1*β* involved in regulating miR-34a transcription. Finally, our investigation shows that IL-1*β* induces transcription of miR-34a, which can directly target the 3′UTR of LGR4, further promoting NF-*κ*B phosphorylation and the inflammatory response [48].

In conclusion, IL-1*β* acts as an agonist of miR-34 transcription, mediates the increased expression of miR-34a, further suppresses LGR4 expression levels, promotes the activity of phosphorylated p65, and triggers the secretion of numerous proinflammatory cytokines to induce an excessive inflammatory response.

## 4. Conclusion

In conclusion, our study provides the first evidence for a novel mechanism of miR-34a augmenting the inflammatory response by triggering the IL-1*β*/LGR4/NF-*κ*B feedforward loop via LGR4 in BENDs. Thus, inhibition of miR-34a may be a novel therapeutic method for protection against endometritis.

## 5. Materials and Methods

### 5.1. Chemical Reagents and Antibodies

Bovine monoclonal antibodies against LGR4 (1 : 1000) were purchased from LifeSpan BioSciences, Inc. (Seattle, WA, USA). Antibodies against NF-*κ*B p65 (1 : 2000), phospho-NF-*κ*B p65 (p-p65) (1 : 2000), and *β*-actin (1 : 3000) and horseradish peroxidase (HRP) goat anti-rabbit (1 : 4000) and goat anti-mouse (1 : 4000) antibodies were obtained from Cell Signalling Technology (Beverly, MA, USA). Sigma-Aldrich (St. Louis, MO, USA) provided foetal bovine serum (FBS) and LPS (*E. coli* 055:B5). Recombinant bovine IL-1*β* protein from Abcam (Ab88013, Cambridge, MA) was used in the study. All other chemicals were reagent grade.

### 5.2. Animal and Tissue Collection

The bovine uterus tissues were collected from local slaughterhouses (Wuhan, China) and farms with Holstein dairy herds (Shiyan, China). Healthy cows from the slaughterhouse were selected for normal uterine samples (*n* = 12). Culled cows on the farm that were clinically monitored for signs of puerperal disease and diagnosed with endometrial inflammatory symptoms by visual inspection, rectal palpation, and vaginal examination (*n* = 8) were targeted for sample collection in the inflammation group. Additionally, endometrium was collected from both uterine horns as described in detail subsequently.

Kunming mice (6 ~ 8 weeks old, 19-22 g weight) (*n* = 24) were purchased from the Experimental Animal Center of Huazhong Agricultural University (Wuhan, China). The mice were kept at room temperature for 12 h in dark-light cycles with free access to food and water. For the detailed treatment of mice see Section 2.8 and Figure 9(a). All procedures followed the guidelines provided by the Laboratory Animal Research Center of Hubei Province and authorized by the Huazhong Agricultural University Animal Care and Use Committee (HZAUMO-2015-12).

### 5.3. Cell Culture

A bovine endometrial epithelial cell line (BEND) and a human embryonic kidney cell line (HEK293T cells) were purchased from the American Type Culture Collection (ATCC TIB-71™). Both cell lines were cultured in Dulbecco's modified Eagle's medium (DMEM, high glucose) supplemented with 10% FBS, 100 U/ml penicillin, and 100 *μ*g/ml streptomycin at 37°C in 5% CO_2_.

### 5.4. MicroRNA Mimic/Inhibitor and siRNA Transfection

Cells (1.5 × 10^5^/ml) were propagated into 96-well plates until the density was approximately 60%-70%, and then 200 nM miR-34a mimic, inhibitor, or their respective negative control duplexes (GenePharma, Shanghai, China) (sequences are shown in S-Table 2) were transfected into BENDs using Lipofectamine™ 2000 (Invitrogen, Carlsbad, California, USA). After 24 h of incubation in the incubator, transfection efficiencies were validated by either qRT-PCR or western blot analysis. Each experiment was repeated three times independently.

### 5.5. Gram Staining and Typan Blue Staining

Bacteria were obtained from endometrial tissue from the uterine horns of cows clinically diagnosed with endometritis or sepsis and cultivated on blood agar. The colonies acquired after 24 hours of incubation were passaged. To characterize the bacterium, Gram staining was conducted on the colonies from the passaged cultures [49] following kit (Solaibao, CAS No. G1060) protocol.

BENDs were removed from the culture plates by trypsinization and mixed with 0.4% Trypan Blue staining solution (Solaibao, CAS No. C0040) at a 9 : 1 dilution, and the percentage of viable cells was determined immediately within 3 min. Cell viability rate (%) = total number of live cells/(total number of live cells + total number of dead cells) × 100%.

### 5.6. Histological Analysis of H&E Staining

After washing with physiological saline, the tissues were immediately fixed in 4% paraformaldehyde for 24 hours. The fixed tissues were embedded in paraffin, cut into 4-micron thickness with a microtome, dehydrated with alcohol (100%, 95%, and 90%) in stages, and stained with hematoxylin and eosin (H&E). The cells were then observed and photographed under an optical microscope (Olympus Shinjuku-ku, Tokyo, Japan). Finally, the pathological activity was evaluated and scored based on the classical pathological scoring system [50–52].

### 5.7. Immunohistochemistry (IHC) Assays

The detailed procedures for tissue fixation, paraffin embedding, and sectioning were the same as those for the H&E staining described in Section 5.6. Sections were deparaffinized with xylene in water and incubated with 3% H_2_O_2_ for 10 min at room temperature, followed by blocking with normal goat serum for 30 min at 37°C. Primary antibodies were incubated overnight at 37°C, and secondary antibodies were incubated for 1 h. After DAB colour development, the sections were counterstained with hematoxylin and observed under a microscope.

### 5.8. Cell Counting Kit (CCK-8) Assay

To detect cell viability after treatment with LPS (1.0 *μ*g/ml) or recombinant IL-1*β* (5 *μ*g/ml), a Cell Counting Kit-8 (CCK-8) assay kit (Beyotime, Shanghai, China) was used. Briefly, the cells (4.5 × 10^4^ cells) were seeded in a 96-well plate and stimulated with LPS or IL-1*β* for 6 h. Then, 10 *μ*l CCK-8 reagent was added and incubated for 3 h at 450 nm to measure the OD value with a microplate reader (Thermo Fisher Scientific, Multiskan MK3, USA).

### 5.9. Flow Cytometry

The effect of LPS (1.0 *μ*g/ml) treatment BENDs on apoptosis and cell cycle was measured by flow cytometry. For cell apoptosis and necrosis, digested cells were stained by Annexin V-FITC and propidium iodide (Annexin V-PI) at 25°C for 15 min according to the manufacturer's instructions (Multisciences Biotech Co., Ltd., Hangzhou, China). For cell cycle assay, BENDs were harvested by trypsin digestion at the indicated time points (0 h, 6 h, 9 h, and 12 h) and fixed in ethanol at -20°C. Cells were then rehydrated and PI stained for 30 min at room temperature in the dark. Stained cells were examined using a flow cytometer (BD Biosciences, USA), and data were optimized using FlowJo software (Tree Star, USA).

### 5.10. Immunofluorescence (IF) Assay

Slides were fixed in 4% paraformaldehyde for 20 min and then incubated in 0.5% Triton X-100 for 20 min. Normal goat serum (10%) was added to permeabilize the cells and block interaction with nonspecific proteins for 30 min, after which antibodies against p-p65 (1 : 1000) and LGR4 (1 : 500) were incubated for 12 h. The fluorescent antibody and DAPI were incubated in a dark box for 1 h and 5 min, respectively. The slides were observed under a fluorescence microscope.

### 5.11. Bioinformatics Analysis

The possible microRNAs for LGR4 were predicted with four algorithms from miRcode (http://www.mircode.org/), miRDB (http://mirdb.org/), TargetScan (http://www.targetscan.org/vert_72/), and ENCORI (http://starbase.sysu.edu.cn/). Then, the overlap of the prediction results of these four websites was determined by Venny (https://bioinfogp.cnb.csic.es/tools/venny/index.html).

### 5.12. Western Blot Assay

Cells and tissues were lysed by RIPA (Biosharp, China) on ice for 30 min, followed by extraction of the total protein. The protein concentration was measured by a BCA kit (Thermo Fisher Scientific, MA, USA) according to the manufacturer's protocols. Then, protein loading buffer was added at a 4 : 1 ratio for denaturation at 95°C for 10 min. Equal amounts of cell lysate proteins were separated by 10% SDS-PAGE and transferred onto PVDF membranes (Millipore, USA). A solution of five percent skimmed milk was used to block the membranes for 2 h and incubated with the primary antibodies (all 1 : 1000 dilution) at 4°C overnight. The bands were incubated with the secondary antibody (1 : 4000 dilution) for 2 h, and the proteins were analysed using ImageJ gel analysis software.

### 5.13. RNA Extraction and qRT-PCR Analysis

Total RNA was extracted from uterine tissues and cells using TRIzol (Invitrogen, USA). The PrimeScript RT Reagent Kit and the miRNA Reverse Transcription System TaqMan MicroRNA Assay were purchased from Applied Biosystems (Foster City, USA). Reverse transcription and quantification of total RNA and miRNA were performed as previously described [53]. Data were normalized to levels of U6 or GAPDH. All primers are shown in S-Tables 1–3.

### 5.14. Plasmid Constructs and Luciferase Reporter Assay

Amplification of the LGR4 3′UTR was performed, and wild-type or mutant-type LGR4 3′UTR plasmids were inserted into a psiCHECK™-2 reporter vector (Promega, Madison, WI, United States) using Xho I and Not I to construct psi-WT-LGR4-3′UTR (WT) and psi-MUT-LGR4-3′UTR (MuT), respectively, which were cotransfected with miR-34a mimics or a negative control into 293T cells using Lipofectamine 2000™. The fluorescence activity was assessed by a dual-luciferase reporter assay system (Promega, Madison, WI, USA). The rate of firefly luciferase activity to Renilla luciferase activity served as the relative luciferase activity.

### 5.15. Statistical Analysis

Results are expressed as the mean ± SEM. The statistical significance of the differences between various treatments was determined by either the two-tailed Student *t*-test or the one-way ANOVA with Bonferroni's posttest, with a probability value of <0.05 considered statistically significant. Data analyses were performed using GraphPad Prism software version 8.0. All experiments were repeated three times.

## Data Availability

The data of this study are available from the corresponding author upon request.

## Supplementary Materials

Supplementary 1 Figure S1: percentage of viable cells. BEND cells were stimulated with LPS (1.0 *μ*g/ml) for 0 h, 3 h, 6 h, 9 h, and 12 h. The trypan blue exclusion test was performed to determine the cell viability by light microscopy. Data are expressed as the mean ± SEM of three independent experiments. A single field of view from a single experiment is presented. Figure S2: detection of cell viability after treatment of BENDs with LPS (1.0 *μ*g/ml) at different time points (0 h, 6 h, 9 h, and 12 h). The cell apoptosis and cell cycle were evaluated by flow cytometry. Data are expressed as mean ± SEM of three independent experiments. ^∗^*P* < 0.05. Figure S3: gram staining for identification of bacteria isolated from the endometrium of cows (*n* = 8). Gram-negative bacteria are light red and Gram-positive bacteria are purple-blue.

Supplementary 2 Table S1: all abbreviations in the study. Table S2: primers for miR-34a and LGR4 siRNA. Table S3: primers used for qRT-PCR. Table S4: histological activity scores in bovine endometritis.
