# Supplementary material for: Enhanced Expression of miR-34a Enhances Escherichia coli Lipopolysaccharide-Mediated Endometritis by Targeting LGR4 to Activate the NF-κB Pathway
Source: Oxid Med Cell Longev. 2021 Aug 27;2021:1744754. doi: 10.1155/2021/1744754 (PMC8422159; doi:10.1155/2021/1744754)

**
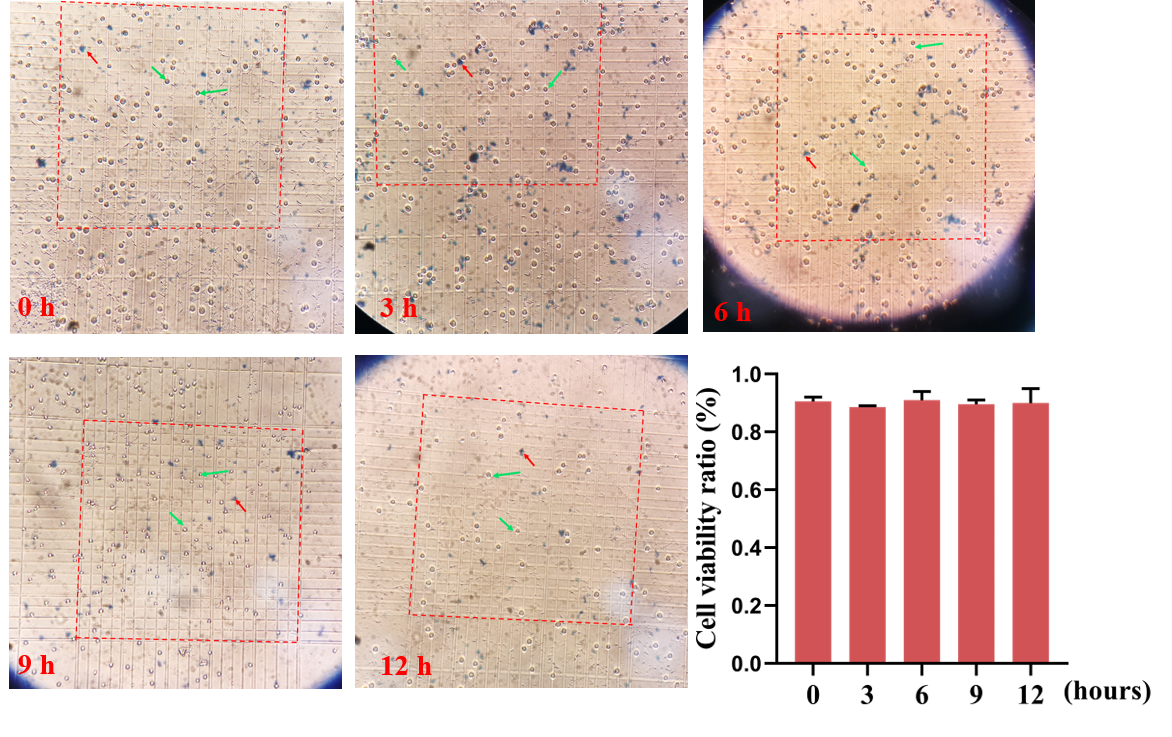
Supplementary materials**

**Fig S1.** Percentage of viable cells. BEND cells were stimulated with LPS (1.0 µg/mL) for 0 h, 3 h, 6 h, 9 h, 12 h. The trypan blue exclusion test was performed to determine the cell viability by light microscopy. Data are expressed as the mean ± SEM of three independent experiments. A single field of view from a single experiment is presented.


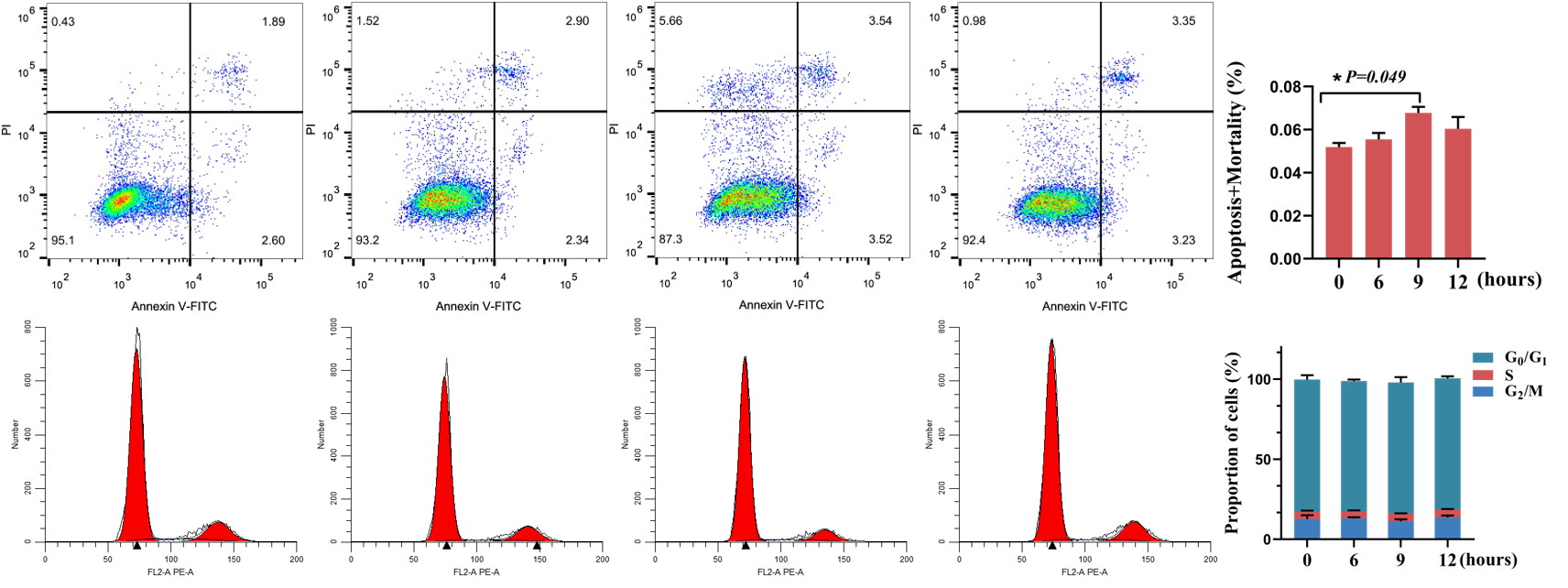


**Fig S2**. Detection of cell viability after treatment of BENDs with LPS (1.0 µg/mL) at different time points (0 h, 6 h, 9 h, 12 h). The cell apoptosis and cell cycle were evaluated by flow cytometry.  Data are expressed as mean ± SEM of three independent experiments. **p* < 0.05.

**
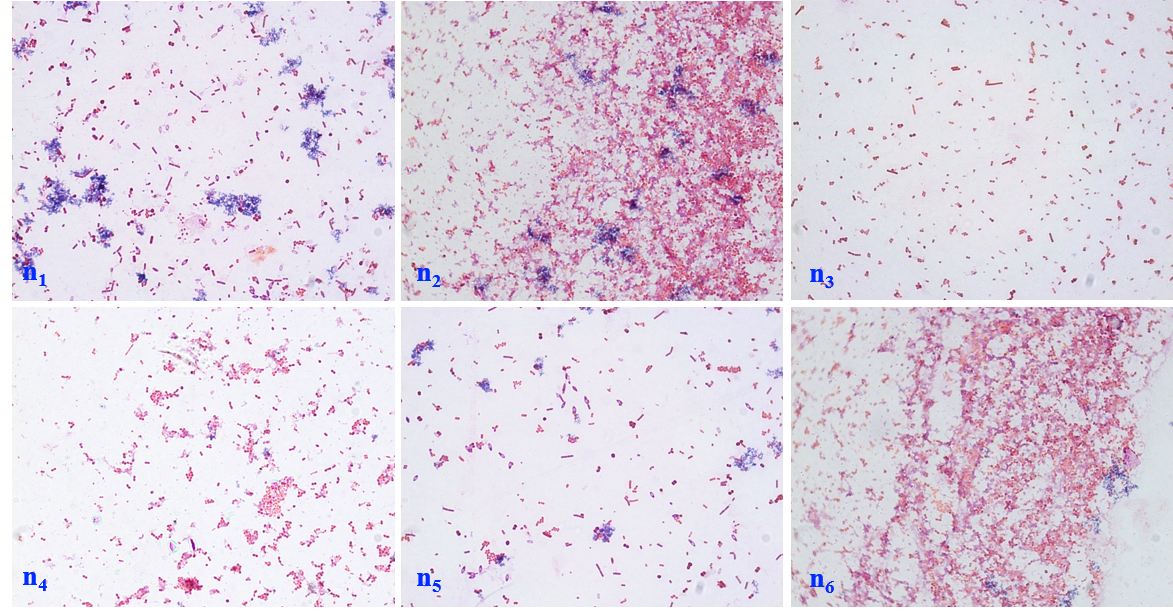
Fig S3.** Gram staining for identification of bacteria isolated from the endometrium of cows (n=8). Gram-negative bacteria are light red and Gram-positive bacteria are purple-blue.

**Gel scans from the main figures**

**Figure 1d**


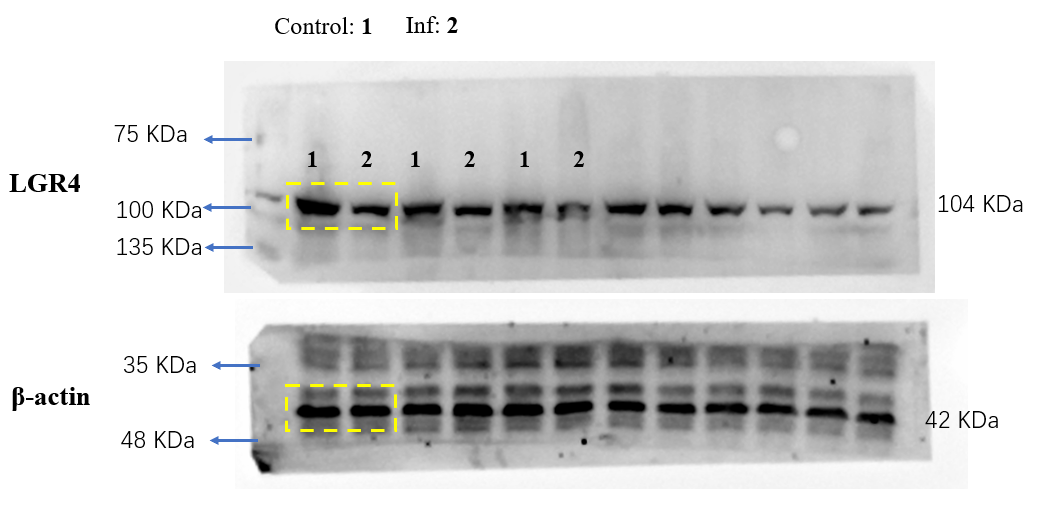


**Figure 2d**


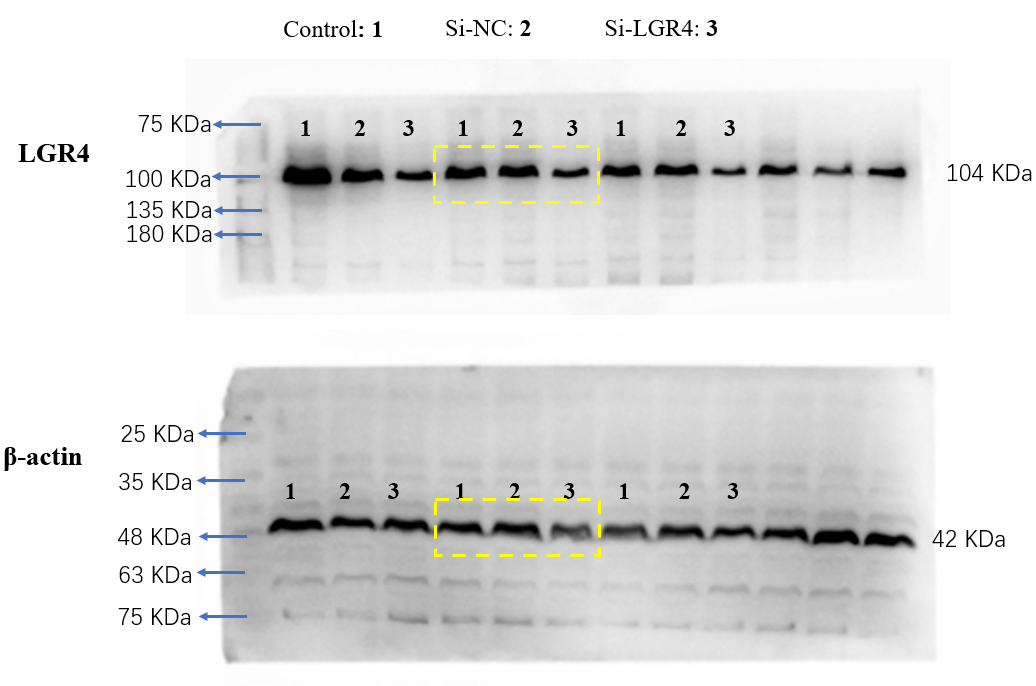


**Figure 3a**


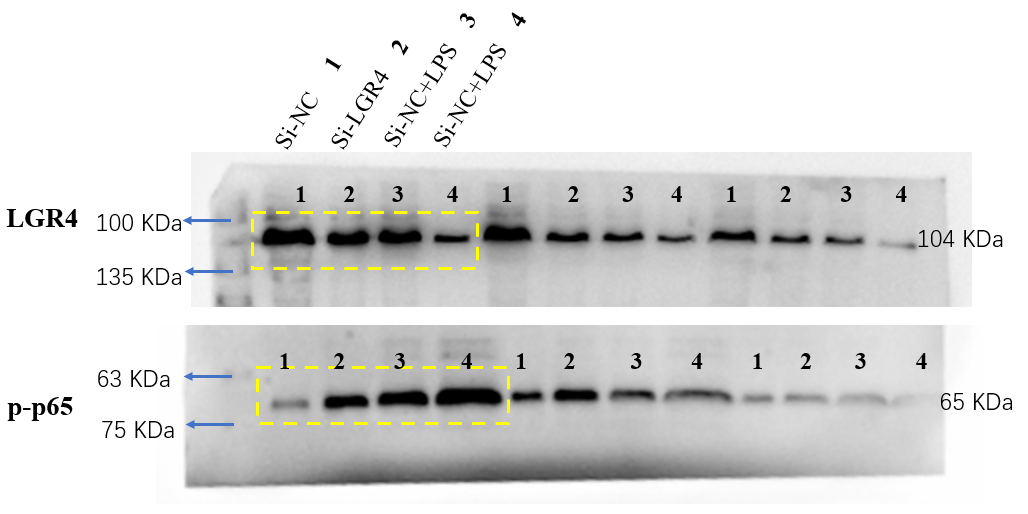


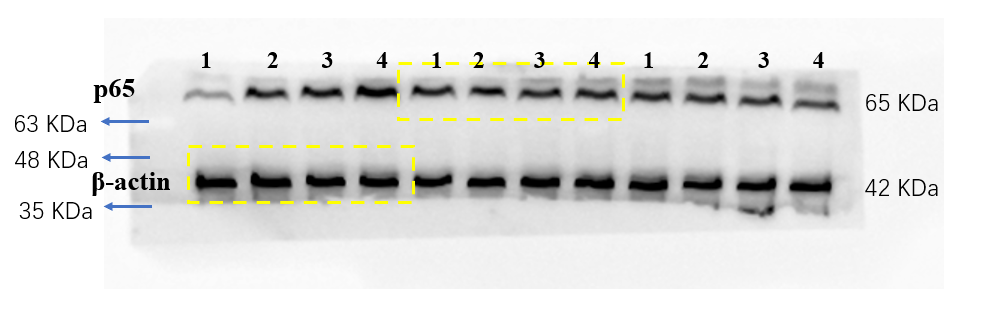


**Figure 4f**


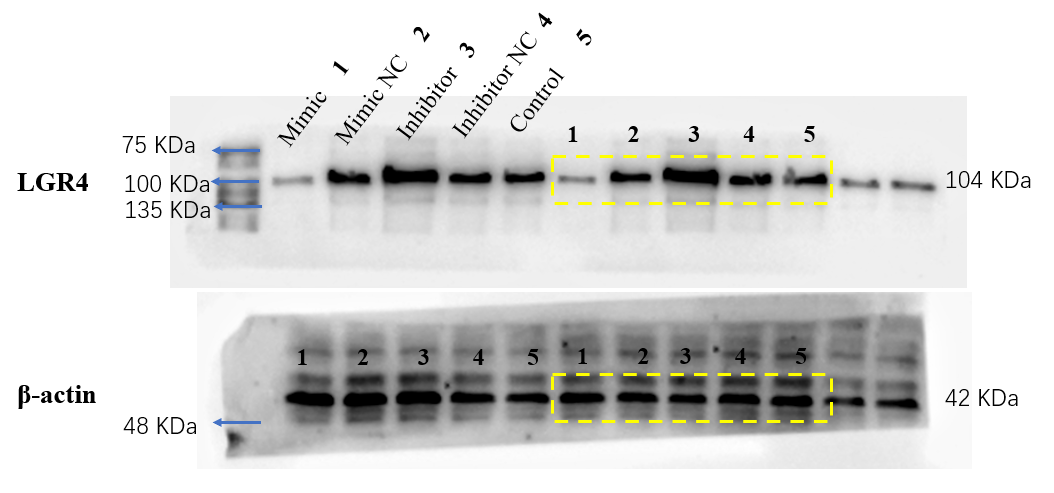


**Figure 6a**


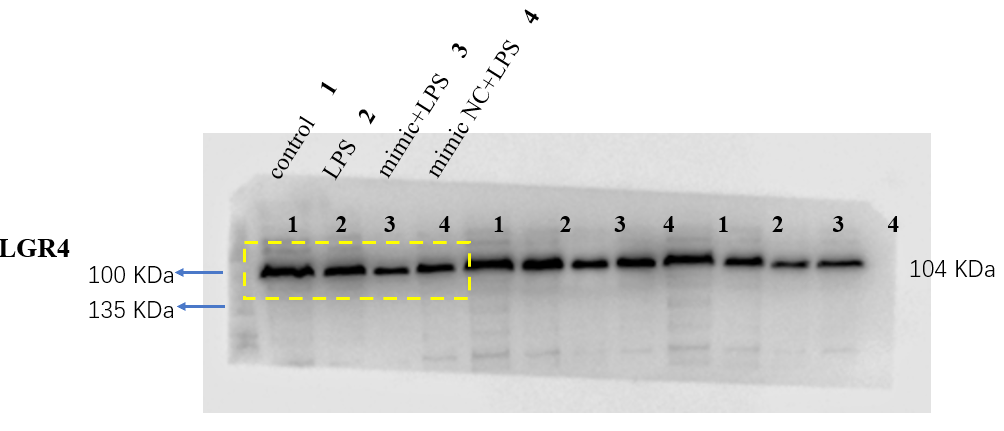


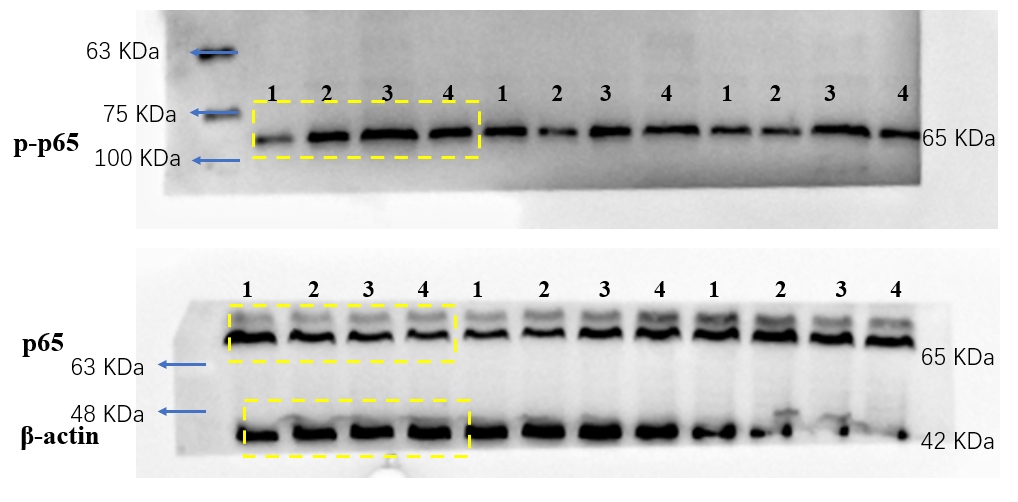


**Figure 7a**


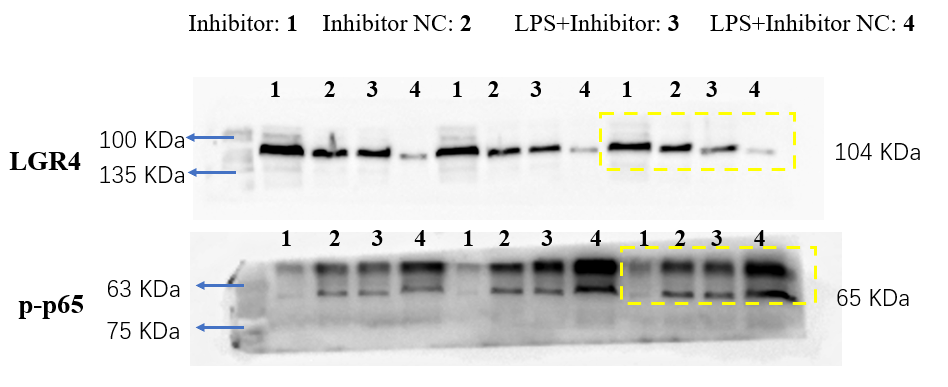


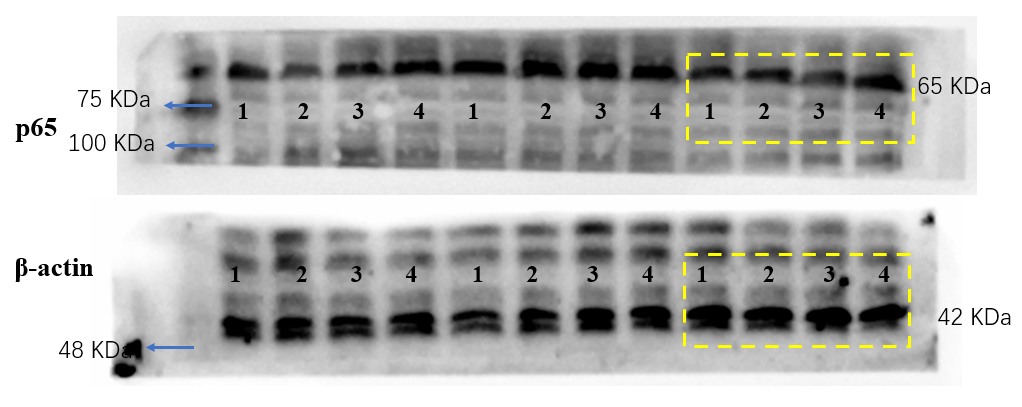


**Figure 8d**


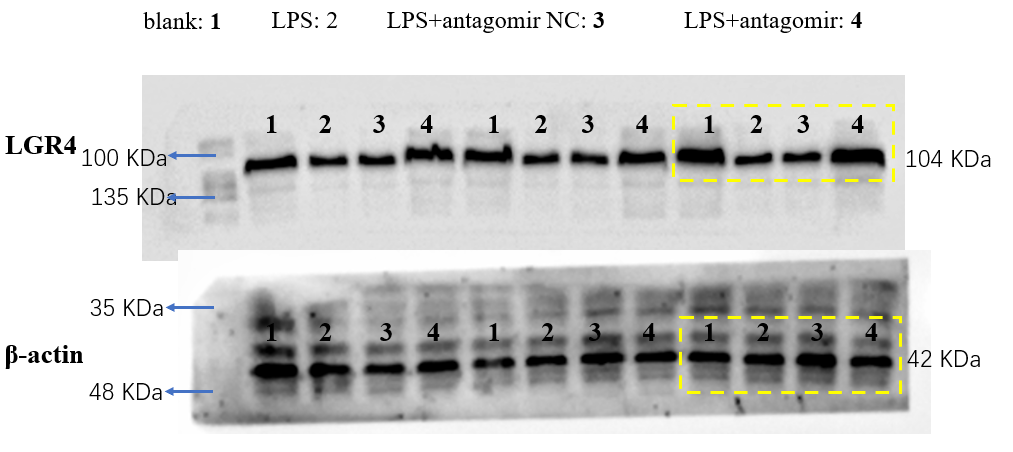


**Figure 9d**


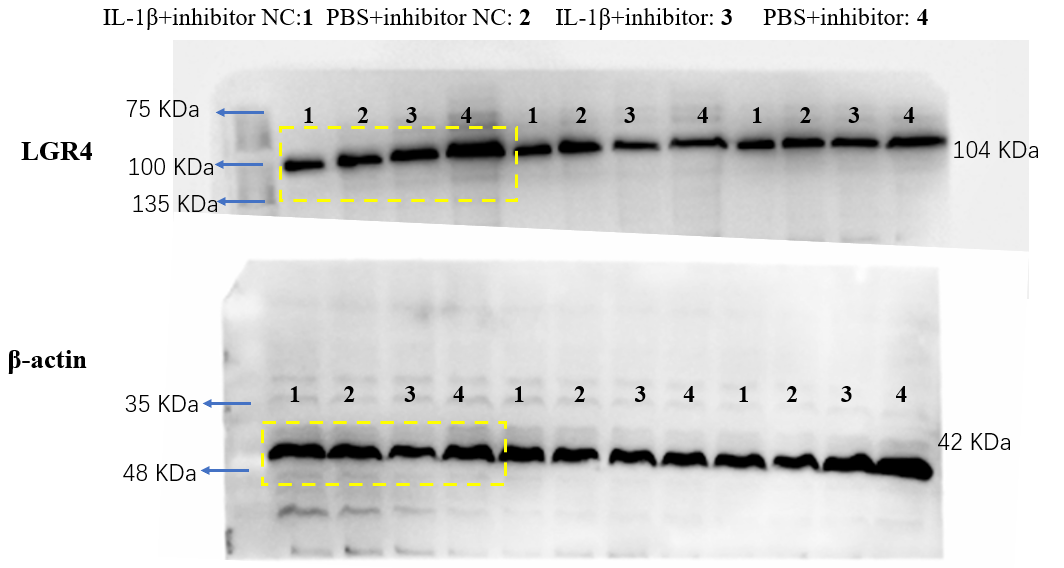

Supplement: Supplementary 1 — Figure S1: percentage of viable cells. BEND cells were stimulated with LPS (1.0 μg/ml) for 0 h, 3 h, 6 h, 9 h, and 12 h. The trypan blue exclusion test was performed to determine the cell viability by light microscopy. Data are expressed as the mean ± SEM of three independent experiments. A single field of view from a single experiment is presented. Figure S2: detection of cell viability after treatment of BENDs with LPS (1.0 μg/ml) at different time points (0 h, 6 h, 9 h, and 12 h). The cell apoptosis and cell cycle were evaluated by flow cytometry. Data are expressed as mean ± SEM of three independent experiments. ∗P < 0.05. Figure S3: gram staining for identification of bacteria isolated from the endometrium of cows (n = 8). Gram-negative bacteria are light red and Gram-positive bacteria are purple-blue. [file 1744754.f1.docx]
