# Supplementary material for: Enhanced Expression of miR-34a Enhances Escherichia coli Lipopolysaccharide-Mediated Endometritis by Targeting LGR4 to Activate the NF-κB Pathway
Source: Oxid Med Cell Longev. 2021 Aug 27;2021:1744754. doi: 10.1155/2021/1744754 (PMC8422159; doi:10.1155/2021/1744754)
Supplement: Supplementary 2 — Table S1: all abbreviations in the study. Table S2: primers for miR-34a and LGR4 siRNA. Table S3: primers used for qRT-PCR. Table S4: histological activity scores in bovine endometritis. [file 1744754.f2.docx]

**Supplementary Table S1-S4.**

**Table S1: All Abbreviations in the study.**

| **Abbr.** | **Full name** |
| --- | --- |
| miRNA | microRNA |
| UTR | Untranslated region |
| LPS | lipopolysaccharide |
| LGR4/Gpr48 | Leucine-Rich Repeat Containing G Protein-Coupled Receptor 4 |
| BEND | Bovine endometrial epithelial cell line |
| NF-κB | Nuclear factor-κ-gene binding |
| IL-1β | Interleukin-1β |
| IL-6 | Interleukin-6 |
| TNF-α | Tumor necrosis factor alpha |
| FBS | Fetal bovine serum |
| qRT-PCR | Quantitative real-time polymerase chain reaction |
| H&E | Hematoxylin and eosin |
| IHC | Immunohistochemistry |
| IF | Immunofluorescene |
| CCK-8 | Cell counting kit-8 |
| TLR2/4 | Toll-like receptor 2/4 |

**Table S2: Primers for miR-34a and LGR4 siRNA.**

| **Gene name** | **Primer sequence (5’-3’)** | **Accession number** |
| --- | --- | --- |
| miR-34a | RT:CTCAACTGGTGTCGTGGAGTCGGCAATTCAGTTGAGACAACCAG | **MI0005464** |
|  | F: CGCGTGGCAGTGTCTTAGCT | [**MIMAT0004340**](http://www.mirbase.org/cgi-bin/mature.pl?mature_acc=MIMAT0004340) |
|  | R: AGTGCAGGGTCCGAGGTATT |  |
| U6 | RT:CGAGCACAGAATCGCTTCACGAATTTGCGTGTCAT | [**NM_001075477.2**](https://www.ncbi.nlm.nih.gov/nuccore/NM_001075477.2) |
|  | F: CGAGCACAGAATCGCTTCA |  |
|  | R: CTCGCTTCGGCAGCACATAT |  |
| mimic | F: TAAGCATGTGGCTTGGCTCA | [**NM_001205511.1**](https://www.ncbi.nlm.nih.gov/nuccore/NM_001205511.1) |
|  | R: AGCAGTCACACACAGTCAGG |  |
| mimic NC | F: GGTGTGTGACGTTCCCATTA | [**NM_174093.1**](https://www.ncbi.nlm.nih.gov/nuccore/NM_174093.1) |
|  | R: ATTGAGGTGGAGAGCTTTCAG |  |
| inhibitor | F: TCCATCCAGTTGCCTTCT | [**NM_173923.2**](https://www.ncbi.nlm.nih.gov/nuccore/NM_173923.2) |
|  | R: TAAGCCTCCGACTTGTGA |  |
| inhibitor NC | F: GTGGAACTGGCAGAAGAG | [**NM_173966.3**](https://www.ncbi.nlm.nih.gov/nuccore/NM_173966.3) |
|  | R: TAAGCCTCCGACTTGTGA |  |
| Si-LGR4 | F: CCACUGCCAUAUUAGUUGUTT | [**NM_001205511.1**](https://www.ncbi.nlm.nih.gov/nuccore/NM_001205511.1) |
|  | R: ACAACUAAUAUGGCAGUGGTT |  |
| antagomir | F: ACAACCAGCUAAGACACUGCCA | [**MIMAT0000542**](http://www.mirbase.org/cgi-bin/mature.pl?mature_acc=MIMAT0000542) |
| antagomir NC | R: CAGUACUUUUGUGUAGUACAA |  |

**Table S3: Primers used for qRT-PCR.**

| **Gene name** | **Primer sequence (5’-3’)** | **Accession number** |
| --- | --- | --- |
| bta-LGR4 | F: TAAGCATGTGGCTTGGCTCA | [**NM_001205511.1**](https://www.ncbi.nlm.nih.gov/nuccore/NM_001205511.1) |
|  | R: AGCAGTCACACACAGTCAGG |  |
| bta-IL-1β | F: GGTGTGTGACGTTCCCATTA | [**NM_174093.1**](https://www.ncbi.nlm.nih.gov/nuccore/NM_174093.1) |
|  | R: ATTGAGGTGGAGAGCTTTCAG |  |
| bta-IL-6 | F: TCCATCCAGTTGCCTTCT | [**NM_173923.2**](https://www.ncbi.nlm.nih.gov/nuccore/NM_173923.2) |
|  | R: TAAGCCTCCGACTTGTGA |  |
| bta-TNF-α | F: GTGGAACTGGCAGAAGAG | [**NM_173966.3**](https://www.ncbi.nlm.nih.gov/nuccore/NM_173966.3) |
|  | R: TAAGCCTCCGACTTGTGA |  |
| bta-GAPDH | F: GGTTGTCTCCTGCGACTTCA | [**NM_001034034.2**](https://www.ncbi.nlm.nih.gov/nuccore/NM_001034034.2) |
|  | R: GGTGGTCCAGGGTTTCTTACT |  |
| mmu-LGR4 | F: AGGGTGTTTGTGAGAGCTGGG | [**XM_036157249.1**](https://www.ncbi.nlm.nih.gov/nuccore/XM_036157249.1) |
|  | R: GGTCGTTACCAGCCAGTTGTA |  |
| mmu-IL-1β | F: GGTGTGTGACGTTCCCATTA | [**XM_006498795.5**](https://www.ncbi.nlm.nih.gov/nuccore/XM_006498795.5) |
|  | R: ATTGAGGTGGAGAGCTTTCAG |  |
| mmu-IL-6 | F: TCCATCCAGTTGCCTTCT | [**NM_001314054.1**](https://www.ncbi.nlm.nih.gov/nuccore/NM_001314054.1) |
|  | R: TAAGCCTCCGACTTGTGA |  |
| mmu-TNF-α | F: GTGGAACTGGCAGAAGAG | [**NM_001278601.1**](https://www.ncbi.nlm.nih.gov/nuccore/NM_001278601.1) |
|  | R: TAAGCCTCCGACTTGTGA |  |
| mmu-GAPDH | F: CAGGTTGTCTCCTGCGACTT | [**NM_001289726.1**](https://www.ncbi.nlm.nih.gov/nuccore/NM_001289726.1) |
|  | R: TATGGGGGTCTGGGATGGAA |  |

**Table S4.**Histological activity scores in Bovine Endometritis

| Features | No grading (0 = absent; 1 = presence) | Grading (scores 0-3) and subscores | | | | Control | Inf |
| --- | --- | --- | --- | --- | --- | --- | --- |
| Ulceration | 0/1 | N/A | | | | 0 | 1 |
| Edema | 0/1 | N/A | | | | 0 | 1 |
|  |  | 0 | 1/3 | 2/3 | 1 |  |  |
| Gland irregularity | N/A | No | Slight | Moderate | Marked | 0 | 1/3 |
| Neutrophil infiltration | N/A | No | Slight | Many foci | Diffuse and heavy | 0 | 1/3 |
| Plasma cell infiltration | N/A | Normal | Excess upper third only | Moderate throughout mucosa | Heavy throught mucosa | 1/3 | 2/3 |
| Iron-containing hemoglobin | N/A | No | Slight | Moderate | Marked | 0 | 1 |
| Cumulative score | | | | | | 1/3 | 13/3 |

Abbreviation: N/A, not applicable.

Description: The pathology score values of the control and Inflammation are marked in red. Control: normal uterus; Inf: inflammatory uterine.
